# Attitudes towards ferric derisomaltose among Swiss patients with iron deficiency and their treating physicians: the prospective, observational Real-CHOICE study

**DOI:** 10.1007/s00404-025-08085-5

**Published:** 2025-06-20

**Authors:** Petra Stute, Pierre-Alexandre Krayenbühl, Stephan R. Vavricka

**Affiliations:** 1 Department of Obstetrics and Gynecology, Inselspital Bern, Friedbuehlstrasse 19, 3010 Bern, Switzerland; 2 Department of Endocrinology, Diabetology and Clinical Nutrition, University Hospital Zurich, Zurich, Switzerland; 3 General Practice Brauereistrasse, Uster, Switzerland; 4 Department of Gastroenterology and Hepatology, University Hospital Zurich, Zurich, Switzerland

**Keywords:** Anemia, Intravenous iron, Switzerland, Treatment satisfaction

## Abstract

**Purpose:**

Iron deficiency (ID) often leads to iron deficiency anemia (IDA). Oral iron supplements are frequently associated with gastrointestinal intolerance and poor adherence. Ferric derisomaltose (FDI), a single high-dose intravenous (IV) infusion, is another option. The real-world Real-CHOICE study investigated the attitudes of patients with IDA and ID without anemia (IDNA) towards IV iron therapy. Secondary objectives were the effectiveness and safety of FDI.

**Methods:**

Real-CHOICE (NCT04318405) enrolled adults with IDA or IDNA in Switzerland. Custom questionnaires evaluated attitudes and treatment satisfaction at baseline and follow-up (≥ 12 weeks). Hemoglobin, ferritin, and transferrin saturation levels, and treatment-emergent adverse events (TEAEs) were assessed.

**Results:**

Of 319 patients (114 with IDA, 205 with IDNA) who received FDI, most were female (n = 305, 93.9%) and aged < 65 years (n = 298, 91.7%). Over three-quarters of patients were not hesitant to receive IV iron treatment at baseline (n = 228, 76.3%) and follow-up (n = 194, 78.2%). The proportion of patients whose attitude towards receiving IV iron therapy remained stable or improved at follow-up versus baseline was 86.1% (n = 204/237). Of 195 patients with available data, mean (standard deviation) changes from baseline to follow-up in hemoglobin, ferritin, and transferrin saturation levels were 1.15 (1.50) g/dL, 144.87 (131.77) µg/L, and 8.71% (13.29%), respectively. TEAEs and adverse drug reactions occurred in 76 (23.8%) and 22 patients (6.9%), respectively.

**Conclusion:**

Real-CHOICE revealed favorable attitudes of patients and physicians towards IV iron therapies, particularly FDI, in a real-world setting. Improved hematologic parameters and favorable safety highlight FDI’s potential as a clinically effective treatment for ID and IDA.

**Supplementary Information:**

The online version contains supplementary material available at 10.1007/s00404-025-08085-5.

## What does this study add to the clinical work

**Table:** 

This real-world study in Switzerland reports positive attitudes and high rates of treatment satisfaction associated with IV iron and FDI in patients with ID and their physicians over ≥ 3 months of follow-up. FDI also demonstrated good effectiveness and safety, suggesting that it is a valuable treatment option for patients with ID.	

## Introduction

Iron deficiency (ID) is a widespread disorder that is typically caused by heavy menstrual bleeding in premenopausal women or by gastrointestinal blood loss in men and postmenopausal women [1]. Other common causes include increased iron need, insufficient dietary iron intake, inadequate iron absorption, or chronic conditions (e.g. chronic kidney disease [CKD] or heart failure) [1]. ID can negatively affect functional outcomes and result in iron deficiency anemia (IDA), which affects approximately 1.27 billion people worldwide [1].

Orally administered iron formulations (e.g., ferrous sulphate) are the first-choice treatment in patients with ID [2], but are associated with gastrointestinal adverse events (AEs) and poor treatment adherence [3]. Intravenous (IV) iron formulations have a better gastrointestinal tolerability profile than oral iron therapies [4]. Modern IV formulations employ a carbohydrate shell that is strongly bound to an iron-containing core, thereby slowing the release of elemental iron and allowing relatively high doses to be administered in a single infusion [5].

In Switzerland, three IV iron formulations for infusion are currently available: iron sucrose (IS), ferric carboxymaltose (FCM) and ferric derisomaltose (FDI), previously known as iron isomaltoside [5]. Because of the strong bond between iron and the derisomaltose matrix, FDI can be administered as a single-dose infusion of up to 20 mg/kg [6]. FDI is available in many countries worldwide, including the United States and the European Union (EU), and was approved in Switzerland in 2019 for the treatment of ID when oral iron supplements are ineffective or cannot be used, or when there is a clinical need for rapid iron supplementation [6]. The safety and efficacy of FDI have been established in randomized clinical trials in patients with IDA of different causes [7–14].

The primary objective of the Real-CHOICE study was to investigate the attitudes of patients with IDA and ID without anemia (IDNA) towards IV iron therapy in the real-world clinical setting in Switzerland. Key secondary objectives were to evaluate the real-world effectiveness and safety of FDI.

## Methods

### Study design

This multicenter, prospective, longitudinal, observational study, conducted across Switzerland, was designed to reflect use of FDI according to the Swiss Summary of Product Characteristics (SmPC) [6]. The decision to use a single FDI administration was made before and independently from inclusion of each patient in the study.

Data were collected during a single FDI administration in routine clinical practice and approximately 12 weeks later; the study design imposed no visit schedule (Supplementary information). Possible re-treatments or splitting of the total dose were beyond the study scope.

### Patients

Legally capable individuals of either sex aged ≥ 18 years were eligible for inclusion if they had been prescribed FDI according to the Swiss SmPC and provided written informed consent for pseudonymized data collection. The key exclusion criterion was prior IV iron treatment or transfusion within 3 months before enrolment.

### Study outcomes

Patients and physicians completed a questionnaire about attitudes towards IV iron and FDI and a treatment satisfaction questionnaire within 30 min after the IV iron infusion, to capture immediate post-infusion impressions, and at the 12-week follow-up visit.

The primary study outcome was the proportion of patients with stable or improved attitudes towards IV iron therapy. Secondary outcomes included patients’ attitudes towards FDI treatment; physicians’ attitudes towards FDI treatment and IV iron treatment, and patients’ and physicians’ treatment satisfaction with FDI; details of iron therapy administered before FDI; reasons for choosing FDI; and patients’ iron needs and FDI dose. Other endpoints included changes in hemoglobin, serum ferritin, and transferrin saturation following FDI treatment; the proportion of patients with IDA with an increase of ≥ 1 g/dL in hemoglobin at follow-up versus baseline; the proportion of patients with IDNA who maintained their baseline hemoglobin level or had a level above the lower limit of normal (LLN) at follow-up versus baseline; and safety and tolerability of FDI (the percentage of patients with AEs or adverse drug reactions [ADRs]; Supplementary information). Pregnancy or maternal exposure during pregnancy were counted as treatment-emergent AEs (TEAEs) in this study, although they are not indicative of drug-related harm. Off-label use was also counted as an AE because it did not follow the labelled indication for FDI.

### Statistical methods

The total population (TP) included all patients who provided informed consent and entered the study. The analysis population (AP) included all patients from the TP who received FDI. The effectiveness population (EP) included all patients from the AP with hemoglobin values.

The target sample size was 450 patients, to be recruited from 70 clinical sites. This calculation assumed that 35% of patients would have stable or improved attitudes at follow-up versus baseline, using a cluster sampling design with precision of d = 6.2%.

Data were summarized using descriptive statistics, including mean, standard deviation (SD), median, and interquartile range (IQR) for continuous variables, and frequencies and percentages for categoric variables. Changes from baseline in hematological parameters were evaluated using a non-parametric signed rank test. Data were analyzed using SAS version 9.4 (SAS Institute Inc., Cary, North Carolina, USA).

## Results

### Patient disposition

**Fig. 1:**
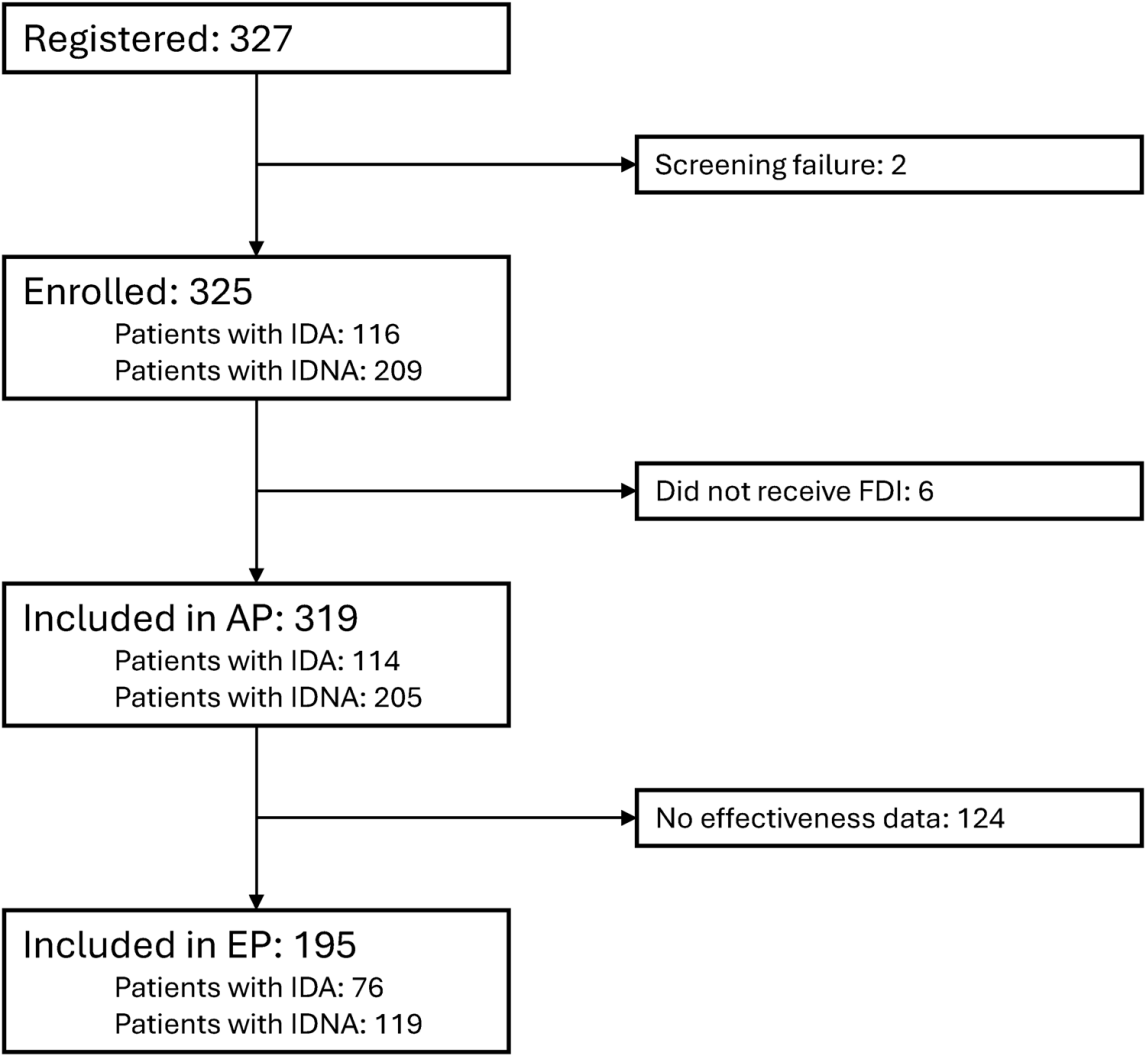
Patient disposition. AP, analysis population; EP, effectiveness population; FDI, ferric derisomaltose; IDA, iron deficiency anemia; IDNA, iron deficiency without anemia

Between 9 July 2020 and 8 September 2022, 327 patients were enrolled at 21 sites (Fig. 1). The TP comprised 325 patients, while the AP comprised 319, of whom 114 had IDA and 205 had IDNA. Of these, 124 patients did not have effectiveness data, resulting in 195 patients in the EP. In the AP, 277 patients (86.8%) completed the study.

Most patients in the AP were female (93.7%) and aged < 65 years (91.5%; Table 1). Median (IQR) body mass index was 23.9 (6.5) kg/m^2^. The IDA versus IDNA group contained fewer females (87.7% vs 97.1%), but more patients aged ≥ 65 years (14.9% vs 4.9%). Most patients were diagnosed with ID at a medical practice (59.6%) or hospital (23.5%). Heavy menstrual bleeding was the most common cause of ID (46.1%).

**Table 1:** Baseline demographic and clinical characteristics, overall and by diagnosis in the analysis population

	IDA (n = 114)	IDNA (n = 205)	Overall (n = 319)
Age, years, median (IQR)	38 (18)	35 (16)	36 (17)
Age, n (%)			
< 65 years	97 (85.1)	195 (95.1)	292 (91.5)
≥ 65 years	17 (14.9)	10 (4.9)	27 (8.5)
Sex, n (%)			
Female	100 (87.7)	199 (97.1)	299 (93.7)
Male	14 (12.3)	6 (2.9)	20 (6.3)
Weight, kg, median (IQR)	67.5 (21.0)	65.0 (16.0)	65.0 (16.0)
BMI, kg/m^2^, median (IQR)	24.9 (7.1)	23.7 (5.6)	23.9 (6.5)
Institution of diagnosis of IDA/IDNA, n (%)			
Medical practice	68 (59.7)	122 (59.5)	190 (59.6)
Hospital	31 (27.2)	44 (21.5)	75 (23.5)
Other	15 (13.2)	39 (19.0)	54 (16.9)
Cause of ID, n (%)	n = 114	n = 205	n = 319
HMB	46 (40.4)	101 (49.3)	147 (46.1)
Pregnancy	6 (5.3)	18 (8.8)	24 (7.5)
CKD	14 (12.3)	8 (3.9)	22 (6.9)
Non-menstrual bleeding	11 (9.7)	2 (1.0)	13 (4.1)
IBD	4 (3.5)	8 (3.9)	12 (3.8)
Malabsorption	5 (4.4)	4 (2.0)	9 (2.8)
Cancer	1 (0.9)	2 (1.0)	3 (0.9)
Other	19 (16.7)	41 (20.0)	60 (18.8)
Not available	8 (7.0)	21 (10.2)	29 (9.1)

*BMI* body mass index, *CKD* chronic kidney disease, *HMB* heavy menstrual bleeding, *IBD* inflammatory bowel disease, *ID* iron deficiency, *IDA* iron deficiency anemia, *IDNA* iron deficiency without anemia, *IQR* interquartile range

Approximately one-third of patients (31.7%) had previously received iron, as oral (16.6%) or IV (17.6%) therapy. Concurrent diseases were present in 136 patients (42.6%), and included hypertension (7.5%), CKD (6.6%), and vitamin D deficiency (3.8%). Forty patients were pregnant at the time of FDI administration.

### FDI treatment

Patients’ iron requirements were most often determined using the simplified FDI dosing scheme (in 70.9% of patients) or the Ganzoni formula (26.7%). Five patients (1.6%) received a fixed dose of FDI 1000 mg. The mean (SD) physician-determined iron requirement was 1005.5 (277.9) mg, the mean (SD) theoretical iron requirement based on the simplified FDI dosing scheme alone was 1256.7 (299.0) mg, and the mean (SD) dose of FDI administered was 902.5 (250.9) mg (Table 2).

**Table 2:** Iron requirements and treatment received, overall and by diagnosis in the analysis population

	IDA (n = 114)	IDNA (n = 205)	Overall (n = 319)
*Iron requirement*			
Physician-calculated,^a^ mg, mean (SD)	1077.5 (337.9)	965.5 (229.5)	1005.5 (277.9)
Calculated for this study,^b^ mg, mean (SD)	1381.0 (350.3)	1189.7 (243.3)	1256.7 (299.0)
*FDI treatment*			
Dose, mg, mean (SD)	941.2 (252.0)	881.0 (248.3)	902.5 (250.9)
Mode of administration, n (%)			
Bolus injection	8 (7.0)	28 (13.7)	36 (11.3)
Infusion	106 (93.0)	177 (86.3)	283 (88.7)
Duration of infusion, min, mean (SD)	36.6 (17.6)	37.7 (16.2)	37.3 (16.7)

*FDI* ferric derisomaltose, *IDA* iron deficiency anemia, *IDNA* iron deficiency without anemia, *SD* standard deviation, *SmPC* summary of product characteristics

^a^Calculated based on the simplified dosing table or the Ganzoni formula provided in the Swiss SmPC for FDI, or based on a fixed dose

^b^Calculated based on the simplified dosing table provided in the Swiss SmPC for FDI

The FDI dose varied between specialists, with the 24 nephrologists administering the highest mean [SD] dose (1166.7 [282.3] mg), followed by the 147 gynecologists (951.0 [195.6] mg), and the 62 gastroenterologists (916.1 [184.8] mg), while the 86 general practitioners administered the lowest dose (736.1 [267.9] mg).

**Fig. 2:**
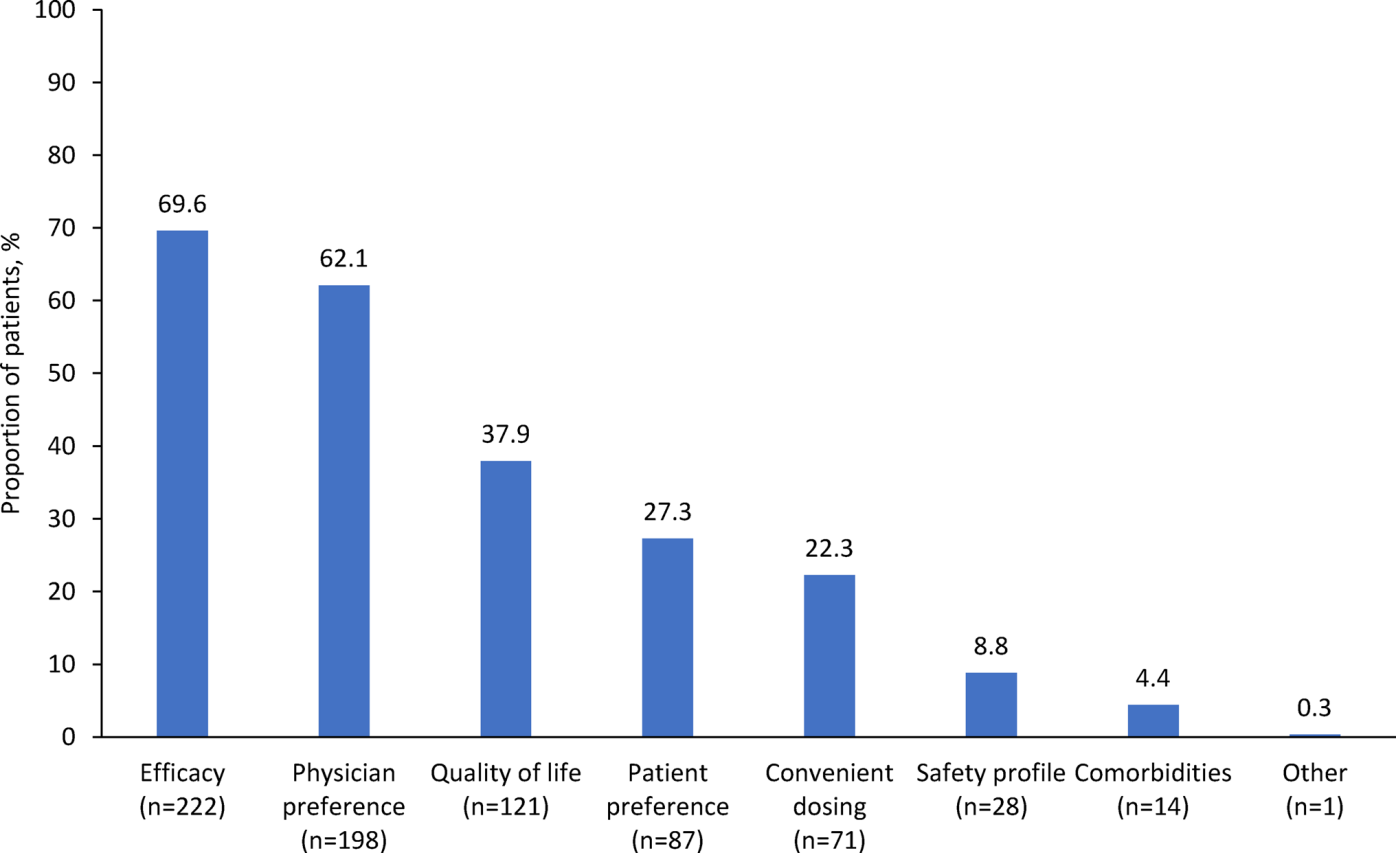
Reasons for choosing ferric derisomaltose

FDI was administered as a bolus injection in 36 patients (11.3%), and as an infusion in 283 patients (88.7%), with a mean (SD) infusion duration of 37.3 (16.7) min. FDI was administered on the day of baseline laboratory evaluation in 10.5% of patients, within 14 days in 54.7%, and > 14 days after baseline laboratory evaluation in 34.4%. The follow-up visit was before week 12 in 60.5% of patients, at week 12 in 12.9%, and after week 12 in 19.4% (7.2% of patients had no follow-up visit). The two most common reasons for choosing FDI were efficacy (69.6%) and physician preference (62.1%; Fig. 2). No subsequent iron therapy was planned in 295 patients (95.8%), most commonly because of FDI treatment success (n = 237, 74.3%), patient decision (n = 35, 11.0%), or physician decision (n = 30, 9.4%).

### Attitudes towards iron therapy

**Fig. 3:**
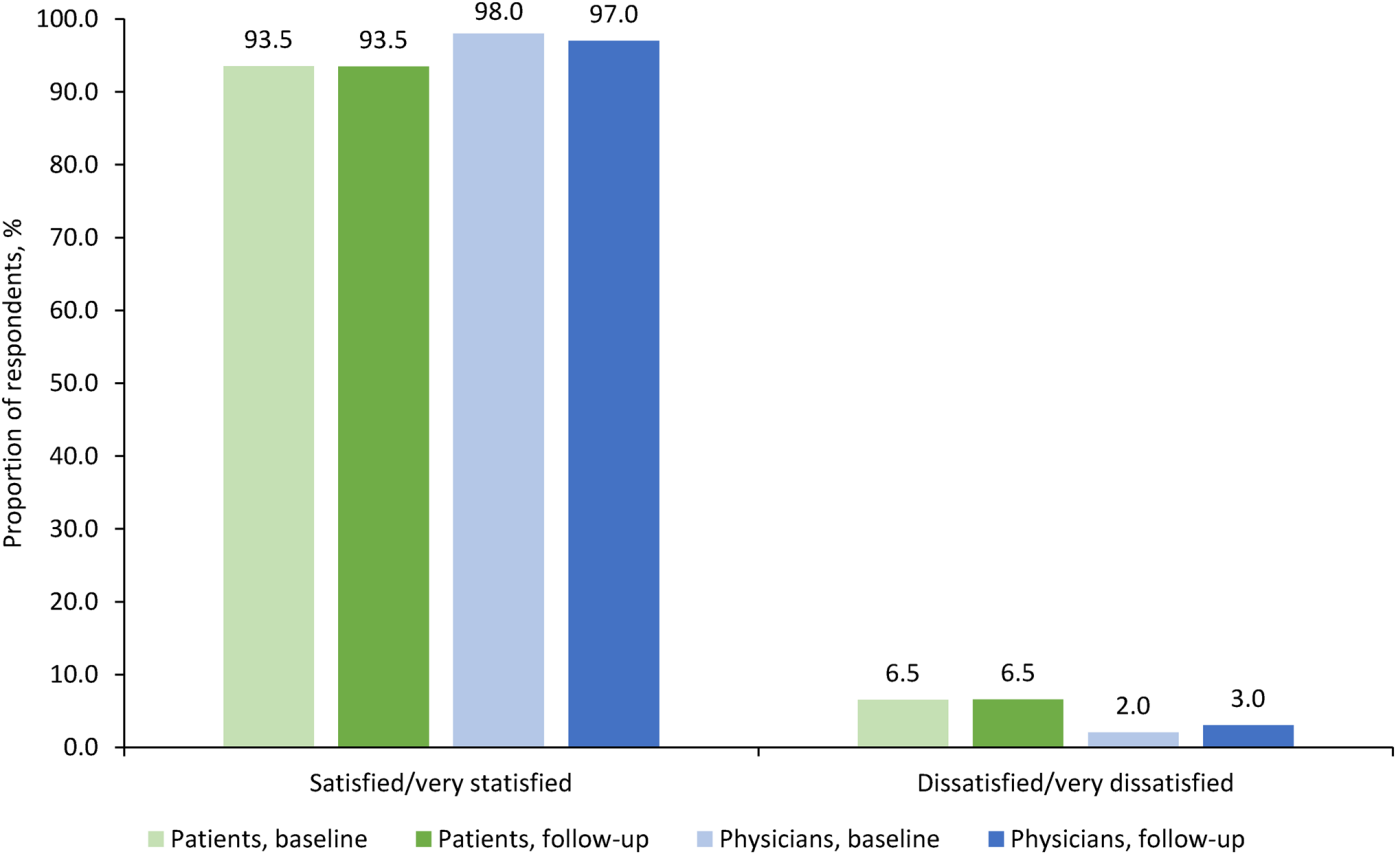
Satisfaction with ferric derisomaltose treatment among patients and physicians. Unanswered or missing questionnaires were not considered; at baseline, 22.9% of patients and 6.3% of physicians did not provide an answer, and at follow-up, 18.5% of patients and 16.9% of physicians did not provide an answer

More than 75% of patients in the AP were not hesitant to receive IV iron at baseline (n/N = 228/299, 76.3%) and follow-up (n/N = 194/248, 78.2%; Supplementary Table S1), and 83–88% of patients mostly or completely agreed with their physician’s choice of FDI treatment (Supplementary Table S2). Overall, 86.1% of patients had a stable or improved attitude towards IV iron (non-hesitancy) at follow-up versus baseline (Table 3). Patient satisfaction with FDI treatment was high and showed no notable change from infusion to follow-up (Fig. 3), remaining stable or improving in 182 patients (90.1%).

**Table 3:** Patients’ and physicians’ attitudes towards IV iron therapy at baseline and follow-up in the analysis population

	Baseline (n = 299)	Follow-up (n = 248)
*Patients’ questionnaire*		
Question 1.1: I am hesitant to be treated with IV iron, n (%)		
Not at all	228 (76.3)	194 (78.2)
Slightly agree	33 (11.0)	31 (12.5)
Mostly agree	12 (4.0)	9 (3.6)
Completely agree	21 (7.0)	13 (5.2)
Not answered	5 (1.7)	1 (0.4)
Missing	20	71
Stability or positive change in attitude of patients towards IV iron (question 1.1),^a^	(n = 237)	
n (%)	204 (86.1)	
*Physicians’ questionnaire*	(n = 299)	(n = 255)
Question 1.1: I am hesitant to treat with IV iron, n (%)		
Not at all	247 (82.6)	208 (81.6)
Slightly agree	19 (6.4)	30 (11.8)
Mostly agree	26 (8.7)	16 (6.3)
Completely agree	7 (2.3)	–
Not answered	–	1 (0.4)
Missing	20	64
Stability or positive change in attitude of physicians towards IV iron (question 1.1),^a^	(n = 246)	
n (%)	224 (91.1)	

*IV* intravenous

^a^Only questionnaires that were answered at both baseline and follow-up were included in this calculation

Physicians’ attitudes towards IV iron and FDI were similar to those of patients (Table 3, Supplementary Tables S3–S4). Treatment satisfaction was high, with 98.0% of physicians satisfied or very satisfied with FDI at baseline, and 97.0% at follow-up (Fig. 3). Satisfaction with FDI remained stable or improved from baseline to follow-up in 223/254 physicians (87.8%).

Subgroup analyses revealed that openness to and satisfaction with both IV iron and FDI were higher in younger patients (aged < 30 years), female patients with ID (mostly due to heavy menstrual bleeding) and fewer concurrent diseases, and lower in older patients (aged ≥ 30 years), including males and females with more concurrent diseases (Supplementary Tables S5–S14).

### Effectiveness

Complete data on hemoglobin were available for 195 patients, on ferritin for 181, and on transferrin saturation for 114 patients in the EP (Table 4). All hematological parameters improved significantly over a median follow-up of 65 days in the EP (P < 0.0001). Starting from a median (IQR) baseline hemoglobin of 10.7 (1.7) g/L, ferritin of 10 (26) μg/L and transferrin saturation of 9.5% (16) in the IDA group, the values increased by + 2.0 (2.5) g/L, + 121 (183) μg/L and + 10% (14.7), respectively. The median (IQR) change from baseline in hemoglobin was 0.70 (1.50) g/dL in the EP, 2.00 (2.45) g/dL in the IDA group, and 0.50 (1.00) g/dL in the IDNA group (Table 4). An increase in hemoglobin of ≥ 1.00 g/dL occurred in 56 of 76 patients (73.7%) in this group.

**Table 4:** Effectiveness of ferric derisomaltose treatment over a median follow-up of 65 days, in the effectiveness population overall and by diagnosis

		n	Median (IQR)	p-value*
*Hemoglobin, g/L*				
IDA	Baseline	76	10.7 (1.7)	
	Follow-up	76	12.9 (1.5)	
	Change from baseline	76	+ 2.0 (2.5)	< 0.0001
IDNA	Baseline	119	12.9 (1.2)	
	Follow-up	118	13.4 (1.3)	
	Change from baseline	118	+ 0.5 (1.0)	< 0.0001
Overall EP	Baseline	195	12.1 (2.3)	
	Follow-up	194	13.2 (1.3)	
	Change from baseline	194	+ 0.7 (1.5)	< 0.0001
*Ferritin, μg/L*				
IDA	Baseline	65	10.0 (26.0)	
	Follow-up	47	132.0 (183.0)	
	Change from baseline	44	+ 121.0 (131.0)	< 0.0001
IDNA	Baseline	116	18.0 (17.8)	
	Follow-up	97	149.0 (114.0)	
	Change from baseline	94	+ 128.0 (115.5)	< 0.0001
Overall EP	Baseline	181	17.0 (21.0)	
	Follow-up	144	144.0 (124.3)	
	Change from baseline	138	+ 125.5 (126.0)	< 0.0001
*Transferrin saturation (%)*				
IDA	Baseline	39	9.5 (16.0)	
	Follow-up	22	25.0 (15.5)	
	Change from baseline	21	+ 10.0 (14.7)	0.0007
IDNA	Baseline	75	21.0 (14.6)	
	Follow-up	41	26.5 (17.7)	
	Change from baseline	39	+ 5.3 (16.0)	0.0012
Overall EP	Baseline	114	17.1 (17.0)	
	Follow-up	63	26.0 (18.1)	
	Change from baseline	60	+ 8.5 (17.3)	< 0.0001

*EP* effectiveness population, *IDA* iron deficiency anemia, *IDNA* iron deficiency without anemia, *IQR* interquartile range

^*^Non-parametric signed rank test

As expected, the increases were smaller in the IDNA group and the total population (Table 4).

### Safety

Overall, 118 TEAEs occurred in 76 patients (23.8%), with similar proportions of IDA (23.7%) and IDNA (23.9%) patients reporting TEAEs (Table 5). Most TEAEs were mild or moderate (National Cancer Institute Common Terminology Criteria for Adverse Events [CTCAE] grade 1 or 2); however, severe TEAEs (grade 3: presyncope and infusion-related reaction) occurred in two patients (0.63%), both in the IDNA group. The most common TEAEs were pregnancy or exposure during pregnancy (12.5%), off-label use (2.8%), headache (1.6%), and Fishbane reaction (1.6%). ADRs causally related to FDI occurred in 22 patients (6.9%); the most common were Fishbane reaction (0.9%), and headache (0.9%).

**Table 5:** Treatment-emergent adverse events and adverse drug reactions in the analysis population

n (%)	IDA (n = 114)	IDNA (n = 205)	Overall (n = 319)
Patients with TEAEs	27 (23.7)	49 (23.9)	76 (23.8)
Grade 3	–	2 (1.0)	2 (0.6)
Patients with ADRs	6 (5.3)	16 (7.8)	22 (6.9)
Grade 3	–	2 (1.0)	2 (0.6)

*ADR* adverse drug reaction, *IDA* iron deficiency anemia, *IDNA* iron deficiency without anemia, *TEAE* treatment-emergent adverse event

Two serious AEs, both considered to be drug-related, occurred in two patients (0.63%) in the IDNA group (Supplementary information). One patient developed grade 2 bronchial obstruction on the day of FDI administration and required hospitalization and treatment. The event resolved without sequelae the following day, and the patient was discharged. The second patient, who was pregnant and had concurrent allergic asthma, developed a grade 3 infusion-related reaction 2–3 min after the start of FDI administration, with respiratory distress, flushing, hard uterus, and fetal bradycardia. The event rapidly resolved without sequelae, following corrective treatment. Two weeks after the event, a healthy infant was delivered by emergency cesarean section.

## Discussion

The Real-CHOICE study shows that, in real-world clinical practice in Switzerland, patients with ID and their physicians have generally positive attitudes towards treatment with IV iron and FDI. Most patients did not express hesitancy towards IV iron therapy at the time of infusion or at follow-up. Physicians consistently showed strong support for IV iron therapy, with most reporting no hesitation at baseline and follow-up. Overall, patients and physicians reported high levels of satisfaction with FDI therapy, which improved mean hemoglobin, ferritin, and transferrin saturation from below-normal at baseline to normal-range at follow-up. FDI therapy also demonstrated a favorable safety and tolerability profile.

After efficacy and physician preference, quality of life was one of the most common reasons for prescribing FDI, cited by physicians as the reason in 37.9% of patients. Previous studies in a range of indications have shown that FDI has a positive effect on quality of life, especially by reducing fatigue [7, 8, 12, 15–18].

Our study identified a discrepancy between physician-determined iron requirements and those calculated using the simplified FDI dosing scheme. There may be several potential reasons for this: our study captured only one FDI dose so may not have captured the full prescribed dose for all patients if more than one infusion was planned; doses may be based on available FDI vial size (e.g., containing 500 or 1000 mg iron); or physicians preferred lower-dose infusions. For these reasons, potential underdosing in the present study must be interpreted with caution. Nevertheless, the lower-than-expected IV iron doses in our study align with results of the prospective, observational NIMO Scandinavian study [19, 20]. The NIMO study also reported that IDA patients who received a higher FDI dose (> 1000 mg vs 1000 mg) had a 65% lower probability of needing FDI re-treatment [19].

In Real-CHOICE, 73.7% of patients with IDA had an increase in hemoglobin of ≥ 1 g/dL, while 98.3% of patients with IDNA maintained hemoglobin at baseline levels or above the LLN. Earlier research on iron therapy has used an increase in hemoglobin of ≥ 2 g/dL to define response [10]. Nonetheless, the 1 g/dL threshold was selected for the current study because we expected to include patients already undergoing iron treatment (with an ongoing increase in hemoglobin), or with hemoglobin maintained in the normal range, and patients requiring chronic iron treatment, who frequently present with non-anemic hemoglobin levels.

Ferritin and transferrin saturation after approximately 12 weeks of follow-up showed notable improvements after FDI, indicating successful iron repletion, which is important to reduce the risk of ID recurrence. In a retrospective study in 117 patients with gastroenterologic disorders and ID, ferritin increased from 26.6 µg/L at baseline to 234.6 µg/L at 1 month after FDI treatment, but fell to 122.8 µg/L at 6 months after FDI treatment. The decrease at 6 months was likely due to blood loss or ongoing iron use during hematopoiesis, because hemoglobin increased throughout the study [21]. These findings underscore the rapidity with which iron depletion can occur in patients with gastrointestinal disorders and ID, and highlight the need for continued monitoring of iron status post-treatment to prevent ID recurrence.

Hypophosphatemia caused by increased fibroblast growth factor 23 (FGF23) is a significant AE following FCM administration, but FDI does not affect FGF23 levels, and is not associated with significant hypophosphatemia [22]. A meta-analysis of 42 prospective clinical studies found that the pooled incidence of hypophosphatemia was 47% after FCM versus 4% after FDI (p < 0.001) [23]. No cases of hypophosphatemia occurred during the present study, but phosphate levels were not routinely monitored and potential delays between infusion and phosphate determination may have influenced our assessment of the hypophosphatemia risk associated with FDI.

A meta-analysis of 15 randomized controlled trials found that the overall risk of serious or severe hypersensitivity reactions was low with modern IV iron formulations and significantly lower with FDI versus FCM (0.14% vs 1.08%; mean odds ratio 0.16 [95% highest posterior density interval 0.05–0.33]) [24]. The low real-world risk of hypersensitivity reactions with FDI was confirmed by a retrospective study in 7354 UK patients, with hypersensitivity reactions occurring in 0.4%, and anaphylactic reactions in < 0.1%, of patients receiving FDI [25]. Two patients (0.63%) in the current study had serious infusion-related reactions, a rate in line with previous studies, and both recovered without sequelae after immediate treatment. Most infusion reactions to IV iron are believed to be caused by complement activation-related pseudo-allergies and slower infusion rates have been associated with lower risk of infusion reactions [26].

The use of IV iron during pregnancy should be preceded by a careful risk–benefit evaluation [27]. Increased vigilance during IV iron administration is also recommended, as there is a risk for transient fetal bradycardia with the use of any IV iron (class effect), which is also noted in the current EU and Swiss SmPCs [6, 28].

Strengths of the Real-CHOICE study are its multicenter, prospective design and real-world setting, thus reflecting the genuine experiences and outcomes of patients undergoing FDI treatment in routine clinical practice in Switzerland. While there is growing evidence regarding the clinical efficacy/safety of IV iron, a novel aspect of Real-CHOICE is the assessment of physician and patient perspectives on this form of treatment. However, the study also had several limitations. First, the COVID-19 pandemic interrupted study recruitment, so we did not meet our goal sample size of 450 patients from 70 clinical sites across Switzerland, despite extending the planned recruitment period. Nevertheless, the primary endpoint result of 86% was higher than expected and had sufficient statistical power. Second, the custom questionnaires used to assess patient and physician attitudes and treatment satisfaction were not externally validated. Third, there was a potential risk of bias related to the timing of the initial post-infusion questionnaire, when patients’ attitudes may have been positively influenced by immediate post-infusion relief and physician reassurance. There was also a risk of selection bias, since the study did not include all ID/IDNA patients, but only those who accepted an IV treatment, excluding patients who declined IV iron. Nevertheless, our data showed that the attitude towards IV iron remained stable between baseline and follow-up in a large proportion of non-hesitant patients. Fourth, the real-world design led to variations in follow-up visits and incomplete laboratory values (e.g. for hematological parameters), complicating uniform assessment of clinical improvements and direct comparisons with other studies. Given the observational nature of the study, comparisons between the ID and IDNA groups were purely descriptive, the lack of inferential testing may have limited the clinical meaning of the results, and (as described above) the observational design may have introduced selection bias. Finally, as this study was conducted in Switzerland, generalizability to other populations may be limited.

In summary, in the Real-CHOICE study, the attitudes of patients with ID and their physicians towards IV iron and FDI remained positive over ≥ 12 weeks of follow-up, mainly showing improvement or no change in hesitancy. Similarly, treatment satisfaction rates were high in patients and physicians. FDI also demonstrated good effectiveness and safety in this real-world setting, with a low risk of serious ADRs, aligned with the results of previous studies. These findings underscore the clinical value of single-dose IV iron for the treatment of IDA and IDNA, including in gynecological settings where these conditions are common.

## Supplementary Information

Below is the link to the electronic supplementary material.

Supplementary file1 (DOCX 95 KB)

## Data Availability

The datasets used and analyzed during the current study are available from the corresponding author on reasonable request.
