# Supplementary material for: Attitudes towards ferric derisomaltose among Swiss patients with iron deficiency and their treating physicians: the prospective, observational Real-CHOICE study
Source: Arch Gynecol Obstet. 2025 Jun 20;312(3):909–18. doi: 10.1007/s00404-025-08085-5 (PMC12374877; doi:10.1007/s00404-025-08085-5)
Supplement: Supplementary file 1 — Supplementary file1 (DOCX 95 KB) [file 404_2025_8085_MOESM1_ESM.docx]

**Attitudes towards ferric derisomaltose among Swiss patients with iron deficiency and their treating physicians: the prospective, observational Real-CHOICE study**

**Supplementary information**

*Archives of Gynecology and Obstetrics*

**Authors:** Petra Stute,^†*^ Pierre-Alexandre Krayenbühl,^†*^ Stephan R. Vavricka

^*^Co-first authors.

^†^These authors contributed equally to this work.

**Corresponding author:** Prof Petra Stute, Department of Obstetrics and Gynecology, Inselspital Bern, Friedbuehlstrasse 19, 3010 Bern, Switzerland. Email: [petra.stute@insel.ch](mailto:petra.stute@insel.ch)

# Supplementary methods and results

### Data collection

Variables collected included demographic data, medical and treatment history, ferric derisomaltose (FDI) administration details, follow-up assessments, and safety variables. All conditions other than iron deficiency (ID) causes were considered concurrent diseases when analyzing medical and treatment histories.

### Adverse events

Adverse events (AEs) and adverse drug reactions (ADRs) were documented and reported according to the Medical Dictionary for Regulatory Affairs. AEs and ADRs were graded according to National Cancer Institute’s Common Terminology Criteria for Adverse Events (CTCAE). Serious AEs (SAEs) were defined as described in the guideline on good pharmacovigilance practices [1]. Definitions of AEs included pregnancy or maternal exposure during pregnancy as special situations, which were counted as treatment-emergent AEs.

### Details of SAEs

Patient 1 was a white, 34-year-old female with ID without anemia (IDNA) who had not previously received iron therapy. Concurrent diseases included Crohn’s disease of the large intestine (since 2015), for which she had received vedolizumab. Within 1–2 min of starting intravenous (IV) FDI, the patient’s blood pressure (BP) was 162/99 mmHg, heart rate was 81 beats per min (bpm), and she developed symptoms of bronchial obstruction (wheezing, dyspnea, desaturation), shivering, nausea, and a sore throat. The patient was hospitalized (July 16–17, 2021) and received twice-daily oral clemastine 2 mg and once-daily oral methylprednisolone 120 mg. After discharge, the patient was prescribed once-daily oral levocetirizine 5 mg and prednisolone 30 mg (July 17–19, 2021).

Patient 2 was a 28-year-old female with IDNA who was 38+3 weeks pregnant and who had previously received oral iron. Concurrent diseases predominantly included allergic asthma and pollen-induced allergic rhinitis, and flatulence and related conditions. Concomitant medication comprised oral simethicone (Flatulex^®^, since March 17, 2022) and a prenatal multivitamin (Natalben Plus^®^, since November 16, 2021). Within 2–3 min of starting the FDI infusion (May 16, 2022), the patient felt her throat swell, she developed dyspnea, stridor, a red facial flush, cold sweat, and a hard uterus; she had a panic reaction. The FDI infusion was terminated. She had oxygen saturation of 92%, BP of 140/80 mmHg, and fetal bradycardia was evident (fetal heart rate 75 bpm). The patient was administered IV clemastine 2 mg and nebulized salbutamol, and was placed on oxygen 4 L/min. After 5 min, the patient reported relief of symptoms. Her oxygen saturation was 98%, and fetal heart rate improved to 120 bpm. The patient recovered completely on the day of the baseline visit and returned home after 1 h. Two weeks after the event, the fetus developed partial respiratory insufficiency and the patient had an emergency caesarean section and delivered a healthy infant. Green amniotic fluid was noted during delivery. The investigator assessed the event of infusion-related reaction to be serious and related to FDI, and categorized it as CTCAE grade 3. The investigator assessed the event of fetal respiratory insufficiency, emergency caesarean section, and green amniotic fluid as not related to FDI.

## Supplementary Table S1

Patients’ attitudes towards intravenous iron treatment at baseline and follow-up in the analysis population

| ***Patients’ attitudes towards IV iron treatment, n (%)*** | **Baseline (n=319)** | **Follow-up (n=319)** |
| --- | --- | --- |
| I am hesitant to be treated with IV iron (question 1.1) |  |  |
| Not at all | 228 (76.3) | 194 (78.2) |
| Slightly agree | 33 (11.0) | 31 (12.5) |
| Mostly agree | 12 (4.0) | 9 (3.6) |
| Completely agree | 21 (7.0) | 13 (5.2) |
| Not answered | 5 (1.7) | 1 (0.4) |
| Missing | 20 | 71 |
| I would consider IV iron treatment due to the physician’s choice (question 1.2) |  |  |
| Not at all | 5 (1.7) | 3 (1.2) |
| Slightly agree | 17 (5.7) | 22 (8.9) |
| Mostly agree | 76 (25.4) | 68 (27.4) |
| Completely agree | 199 (66.6) | 153 (61.7) |
| Not answered | 2 (0.7) | 2 (0.8) |
| Missing | 20 | 71 |
| I would consider IV iron treatment due to its safety compared to other iron treatment options (question 1.3) |  |  |
| Not at all | 21 (7.0) | 7 (2.8) |
| Slightly agree | 53 (17.7) | 46 (18.6) |
| Mostly agree | 47 (15.7) | 51 (20.6) |
| Completely agree | 123 (41.1) | 106 (42.7) |
| Not answered | 9 (3.0) | 3 (1.2) |
| Don’t know | 46 (15.4) | 35 (14.1) |
| Missing | 20 | 71 |
| I would consider IV iron treatment due to its efficacy compared to other iron treatment options (question 1.4) |  |  |
| Not at all | 39 (13.0) | 32 (12.9) |
| Slightly agree | 15 (5.0) | 15 (6.1) |
| Mostly agree | 38 (12.7) | 40 (16.1) |
| Completely agree | 182 (60.9) | 140 (56.5) |
| Not answered | 6 (2.0) | 5 (2.0) |
| Don’t know | 19 (6.4) | 16 (6.5) |
| Missing | 20 | 71 |

IV, intravenous.

## Supplementary Table S2

Patients’ attitudes towards ferric derisomaltose treatment at baseline and follow-up in the analysis population

| ***Patients’ attitudes towards FDI treatment, n (%)*** | **Baseline (n=319)** | **Follow-up (n=319)** |
| --- | --- | --- |
| I am hesitant to be treated with FDI (question 2.1) |  |  |
| Not at all | 228 (76.3) | 185 (74.6) |
| Slightly agree | 26 (8.7) | 28 (11.3) |
| Mostly agree | 12 (4.0) | 10 (4.0) |
| Completely agree | 18 (6.0) | 15 (6.1) |
| Not answered | 15 (5.0) | 10 (4.0) |
| Missing | 20 | 71 |
| I would consider FDI treatment due to the physician’s choice (question 2.2) |  |  |
| Not at all | 5 (1.7) | 9 (3.63) |
| Slightly agree | 24 (8.0) | 29 (11.7) |
| Mostly agree | 73 (24.4) | 64 (25.8) |
| Completely agree | 190 (63.6) | 141 (56.9) |
| Not answered | 7 (2.3 | 5 (2.0) |
| Missing | 20 | 71 |
| I would consider FDI treatment due to its safety compared to other iron treatment options (question 2.3) |  |  |
| Not at all | 17 (5.7) | 9 (3.6) |
| Slightly agree | 54 (18.1) | 48 (19.4) |
| Mostly agree | 48 (16.1) | 40 (16.1) |
| Completely agree | 113 (37.8) | 110 (44.4) |
| Not answered | 11 (3.7) | 5 (2.0) |
| Don’t know | 56 (18.7) | 36 (14.5) |
| Missing | 20 | 71 |
| I would consider FDI treatment due to its efficacy compared to other iron treatment options (question 2.4) |  |  |
| Not at all | 38 (12.7) | 33 (13.3) |
| Slightly agree | 15 (5.0) | 20 (8.1) |
| Mostly agree | 46 (15.4) | 49 (19.8) |
| Completely agree | 140 (46.8) | 123 (49.6) |
| Not answered | 6 (2.0) | 3 (1.2) |
| Don’t know | 54 (18.1) | 20 (8.1) |
| Missing | 20 | 71 |
| I would consider FDI treatment due to its specific dosing and administration schedule (question 2.5) |  |  |
| Not at all | 8 (2.7) | 6 (2.4) |
| Slightly agree | 54 (18.1) | 46 (18.6) |
| Mostly agree | 43 (14.4) | 40 (16.1) |
| Completely agree | 128 (42.8) | 108 (43.6) |
| Not answered | 12 (4.0) | 6 (2.4) |
| Don’t know | 54 (18.1) | 42 (17.0) |
| Missing | 20 | 71 |

FDI, ferric derisomaltose.

## Supplementary Table S3

Physicians’ attitudes towards intravenous iron treatment at baseline and follow-up

| ***Physicians’ attitudes towards IV iron treatment, n (%)*** | **Baseline (n=301)** | **Follow-up (n=301)** |
| --- | --- | --- |
| I am hesitant to treat with IV iron (question 1.1) |  |  |
| Not at all | 247 (82.6) | 208 (81.6) |
| Slightly agree | 19 (6.4) | 30 (11.8) |
| Mostly agree | 26 (8.7) | 16 (6.3) |
| Completely agree | 7 (2.3) | 0 (0.0) |
| Not answered | 0 | 1 (0.4) |
| Missing | 20 | 64 |
| I would consider IV iron treatment due to the patient’s desire (question 1.2) |  |  |
| Not at all | 11 (3.7) | 7 (2.8) |
| Slightly agree | 80 (26.8) | 82 (32.2) |
| Mostly agree | 106 (35.5) | 80 (31.4) |
| Completely agree | 100 (33.4) | 84 (32.9) |
| Not answered | 2 (0.7) | 2 (0.8) |
| Missing | 20 | 64 |
| I would consider IV iron treatment due to its safety compared to other iron treatment options (question 1.3) |  |  |
| Not at all | 36 (12.0) | 27 (10.6) |
| Slightly agree | 101 (33.8) | 77 (30.2) |
| Mostly agree | 65 (21.7) | 73 (28.6) |
| Completely agree | 97 (32.4) | 77 (30.2) |
| Not answered | 0 (0.0) | 1 (0.4) |
| Missing | 20 | 64 |
| I would consider IV iron treatment due to its efficacy compared to other iron treatment options (question 1.4) |  |  |
| Not at all | 43 (14.4) | 36 (14.1) |
| Slightly agree | 6 (2.0) | 5 (2.0) |
| Mostly agree | 50 (16.7) | 46 (18.0) |
| Completely agree | 199 (66.6) | 168 (66.0) |
| Not answered | 1 (0.3) | 0 (0.0) |
| Missing | 20 | 64 |

IV, intravenous.

## Supplementary Table S4

Physicians’ attitudes towards ferric derisomaltose treatment at baseline and follow-up in the analysis population

| ***Physicians’ attitudes towards FDI treatment, n (%)*** | **Baseline (n=301)** | **Follow-up (n=301)** |
| --- | --- | --- |
| I am hesitant to treat with FDI (question 2.1) |  |  |
| Not at all | 236 (78.9) | 178 (69.8) |
| Slightly agree | 30 (10.0) | 48 (18.8) |
| Mostly agree | 15 (5.0) | 24 (9.4) |
| Completely agree | 4 (1.3) | 2 (0.8) |
| Not answered | 14 (4.7) | 3 (1.2) |
| Missing | 20 | 64 |
| I would consider FDI treatment due to the patient’s desire (question 2.2) |  |  |
| Not at all | 8 (2.7) | 3 (1.2) |
| Slightly agree | 71 (23.8) | 75 (29.4) |
| Mostly agree | 106 (35.5) | 82 (32.2) |
| Completely agree | 114 (38.1) | 93 (36.5) |
| Not answered | 0 (0.0) | 2 (0.8) |
| Missing | 20 | 64 |
| I would consider FDI treatment due to its safety compared to other iron treatment options (question 2.3) |  |  |
| Not at all | 29 (9.7) | 24 (9.4) |
| Slightly agree | 104 (34.8) | 85 (33.3) |
| Mostly agree | 66 (22.1) | 68 (26.7) |
| Completely agree | 100 (33.4) | 76 (29.8) |
| Not answered | 0 (0.0) | 2 (0.8) |
| Missing | 20 | 64 |
| I would consider FDI treatment due to its efficacy compared to other iron treatment options (question 2.4) |  |  |
| Not at all | 63 (21.1) | 42 (16.5) |
| Slightly agree | 28 (9.4) | 25 (9.8) |
| Mostly agree | 73 (24.4) | 55 (21.6) |
| Completely agree | 133 (44.5) | 130 (51.0) |
| Not answered | 2 (0.7) | 3 (1.2) |
| Missing | 20 | 64 |
| I would consider FDI treatment due to its specific dosing and administration schedule (question 2.5) |  |  |
| Not at all | 27 (9.0) | 7 (2.8) |
| Slightly agree | 83 (27.8) | 78 (30.6) |
| Mostly agree | 60 (20.1) | 56 (22.0) |
| Completely agree | 127 (42.5) | 113 (44.3) |
| Not answered | 2 (0.7) | 1 (0.4) |
| Missing | 20 | 64 |

FDI, ferric derisomaltose.

## Supplementary Table S5

Mean age of patients in the total population by baseline demographic and clinical characteristics

| **Age, years, mean (SD)** | **(N=325)** |
| --- | --- |
| Diagnosis of ID |  |
| IDA (n=116) | 42.3 (17.9) |
| IDNA (n=209) | 36.8 (13.3) |
| Age group |  |
| 18–30 years (n=113) | 24.9 (3.6) |
| 31–40 years (n=98) | 35.9 (2.9) |
| >40 years (n=114) | 55.0 (13.7) |
| Sex |  |
| Female (n=305) | 37.0 (13.0) |
| Male (n=20) | 66.2 (20.9) |
| Cause of ID |  |
| HMB (n=151) | 34.3 (9.7) |
| Other (n=145) | 41.7 (18.4) |
| Concurrent disease |  |
| Yes (n=143) | 44.4 (18.4) |
| No (n=182) | 34.4 (10.5) |
| Concomitant medication/therapeutic intervention |  |
| Yes (n=117) | 45.9 (19.3) |
| No (n=208) | 34.8 (10.7) |

HMB, heavy menstrual bleeding; ID, iron deficiency; IDA, iron deficiency anemia; IDNA, iron deficiency without anemia; SD, standard deviation.

## Supplementary Table S6

Baseline demographic and clinical characteristics in the total population by sex

| **n (%)** | **Female** | **Male** |
| --- | --- | --- |
| Diagnosis of ID |  |  |
| IDA (n=116) | 102 (87.9) | 14 (12.1) |
| IDNA (n=209) | 203 (97.1) | 6 (2.9) |
| Age group |  |  |
| 18–30 years (n=113) | 110 (97.3) | 3 (2.7) |
| 31–40 years (n=98) | 97 (99.0) | 1 (1.0) |
| >40 years (n=114) | 98 (86.0) | 16 (14.0) |
| Concurrent disease |  |  |
| Yes (n=143) | 125 (87.4) | 18 (12.6) |
| No (n=182) | 180 (98.9) | 2 (1.1) |
| Concomitant medication/therapeutic intervention |  |  |
| Yes (n=117) | 99 (84.6) | 18 (15.4) |
| No (n=208) | 206 (99.0) | 2 (1.0) |

ID, iron deficiency; IDA, iron deficiency anemia; IDNA, iron deficiency without anemia.

## Supplementary Table S7

Patients’ overall treatment satisfaction with ferric derisomaltose at follow-up, and sex of patients in the total population, by diagnosis

| **n (%)** | **IDA** | **IDNA** |
| --- | --- | --- |
| Overall satisfaction with FDI treatment |  |  |
| Very dissatisfied (n=17) | 7 (41.2) | 10 (58.8) |
| Satisfied (n=94) | 42 (44.7) | 52 (55.3) |
| Very satisfied (n=149) | 39 (26.2) | 110 (73.8) |
| Sex |  |  |
| Female (n=305) | 102 (33.4) | 203 (66.6) |
| Male (n=20) | 14 (70.0) | 6 (30.0) |

FDI, ferric derisomaltose; IDA, iron deficiency anemia; IDNA, iron deficiency without anemia.

## Supplementary Table S8

Patients’ overall treatment satisfaction with ferric derisomaltose at follow-up, and baseline demographic and clinical characteristics, by cause of iron deficiency

| **n (%)** | **Cause of ID^a^** | | | | | | | | |
| --- | --- | --- | --- | --- | --- | --- | --- | --- | --- |
|  | **CKD** | **IBD** | **Cancer** | **Bleeding NM** | **HMB** | **Pregnancy** | **Malabsorption** | **Other** | **Unknown** |
| Overall satisfaction with FDI treatment at follow-up |  |  |  |  |  |  |  |  |  |
| Very dissatisfied (n=17) | 1 (5.9) | 0 (0.0) | 0 (0.0) | 0 (0.0) | 3 (17.6) | 2 (11.8) | 0 (0.0) | 8 (47.1) | 3 (17.6) |
| Satisfied (n=94) | 17 (18.1) | 5 (5.3) | 1 (1.1) | 4 (4.3) | 32 (34.0) | 11 (11.7) | 3 (3.2) | 15 (16.0) | 6 (6.4) |
| Very satisfied (n=149) | 4 (2.7) | 5 (3.4) | 2 (1.3) | 7 (4.7) | 84 (56.4) | 5 (3.4) | 6 (4.0) | 23 (15.4) | 13 (8.7) |
| Age group |  |  |  |  |  |  |  |  |  |
| 18–30 years (n=113) | 2 (1.8) | 5 (4.4) | 0 (0.0) | 3 (2.7) | 63 (55.8) | 13 (11.5) | 2 (1.8) | 20 (17.7) | 5 (4.4) |
| 31–40 years (n=98) | 2 (2.0) | 4 (4.1) | 0 (0.0) | 6 (6.1) | 42 (42.9) | 13 (13.3) | 3 (3.1) | 25 (25.5) | 3 (3.1) |
| >40 years (n=114) | 18 (15.8) | 3 (2.6) | 3 (2.6) | 4 (3.5) | 46 (40.4) | 0 (0.0) | 4 (3.5) | 15 (13.2) | 21 (18.4) |
| Sex |  |  |  |  |  |  |  |  |  |
| Female (n=305) | 11 (3.6) | 9 (3.0) | 2 (0.7) | 12 (3.9) | 151 (49.5) | 26 (8.5) | 9 (3.0) | 56 (18.4) | 29 (9.5) |
| Male (n=20) | 11 (55.0) | 3 (15.0) | 1 (5.0) | 1 (5.0) | 0 (0.0) | 0 (0.0) | 0 (0.0) | 4 (20.0) | 0 (0.0) |
| Concurrent disease |  |  |  |  |  |  |  |  |  |
| Yes (n=143) | 22 (18.8) | 12 (8.4) | 3 (2.1) | 10 (7.0) | 43 (30.1) | 17 (11.9) | 5 (3.5) | 16 (11.2) | 15 (10.5) |
| No (n=182) | 0 (0.0) | 0 (0.0) | 0 (0.0) | 3 (1.6) | 108 (59.3) | 9 (4.9) | 4 (2.2) | 44 (24.2) | 14 (12.0) |
| Concomitant medication/therapeutic intervention | | |  |  |  |  |  |  |  |
| Yes (n=117) | 22 (18.8) | 11 (9.4) | 2 (1.7) | 6 (5.1) | 27 (23.1) | 19 (16.2) | 3 (2.6) | 13 (11.1) | 14 (12.0) |
| No (n=208) | 0 (0.0) | 1 (0.5) | 1 (0.5) | 7 (3.4) | 124 (59.6) | 7 (3.4) | 6 (2.9) | 47 (22.6) | 15 (7.2) |

^a^Causes of ID included congestive heart failure (CHF) and all other categories presented in this table, but as there were no patients with CHF, this category has not been included in the table.

CKD, chronic kidney disease; FDI, ferric derisomaltose; HMB, heavy menstrual bleeding; IBD, inflammatory bowel disease; ID, iron deficiency; NM, non-menstrual.

## Supplementary Table S9

Patients’ overall treatment satisfaction with ferric derisomaltose treatment at follow-up, and baseline demographic and clinical characteristics in the total population, by concurrent disease status

|  | **Concurrent diseases** | |
| --- | --- | --- |
|  | **Yes** | **No** |
| Overall satisfaction with FDI treatment at follow-up |  |  |
| Very dissatisfied (n=17) | 6 (35.3) | 11 (64.7) |
| Satisfied (n=94) | 60 (63.8) | 34 (36.2) |
| Very satisfied (n=149) | 58 (38.9) | 91 (61.1) |
| Age group |  |  |
| 18–30 years (n=113) | 40 (35.4) | 73 (64.6) |
| 31–40 years (n=98) | 37 (37.8) | 61 (62.2) |
| >40 years (n=114) | 66 (57.9) | 48 (42.1) |
| Sex |  |  |
| Female (n=305) | 125 (41.0) | 180 (71.5) |
| Male (n=20) | 18 (90.0) | 2 (10.0) |
| Cause of ID |  |  |
| HMB (n=151) | 43 (28.5) | 108 (71.5) |
| Other (n=145) | 85 (58.6) | 60 (41.4) |
| Concomitant medication/therapeutic intervention |  |  |
| Yes (n=117) | 113 (96.6) | 4 (3.4) |
| No (n=208) | 30 (14.4) | 178 (85.6) |

FDI, ferric derisomaltose; HMB, heavy menstrual bleeding; ID, iron deficiency.

## Supplementary Table S10

Patients’ overall treatment satisfaction with ferric derisomaltose treatment at follow-up, and baseline demographic and clinical characteristics in the total population, by concomitant medication or therapeutic intervention status

|  | **Concomitant medication/therapeutic intervention** | |
| --- | --- | --- |
|  | **Yes** | **No** |
| Overall satisfaction with FDI treatment at follow-up |  |  |
| Very dissatisfied (n=17) | 7 (41.2) | 10 (58.8) |
| Satisfied (n=94) | 49 (52.1) | 45 (47.9) |
| Very satisfied (n=149) | 49 (32.9) | 100 (67.1) |
| Age group |  |  |
| 18–30 years (n=113) | 30 (26.5) | 83 (73.5) |
| 31–40 years (n=98) | 33 (33.7) | 65 (66.3) |
| >40 years (n=114) | 54 (47.4) | 60 (52.6) |
| Sex |  |  |
| Female (n=305) | 99 (32.5) | 206 (67.5) |
| Male (n=20) | 18 (90.0) | 2 (10.0) |
| Cause of ID |  |  |
| HMB (n=151) | 27 (17.9) | 124 (82.1) |
| Other (n=145) | 76 (52.4) | 69 (47.6) |
| Concurrent disease |  |  |
| Yes (n=143) | 113 (79.0) | 30 (21.0) |
| No (n=182) | 4 (2.2) | 178 (97.8) |

FDI, ferric derisomaltose; HMB, heavy menstrual bleeding; ID, iron deficiency.

## Supplementary Table S11

Baseline demographic and clinical characteristics of patients by overall level of physician satisfaction with ferric derisomaltose treatment at baseline

| **n (%)** | **Physician satisfaction at baseline** | | | | |
| --- | --- | --- | --- | --- | --- |
|  | **Very dissatisfied** | **Dissatisfied** | **Satisfied** | **Very satisfied** | **Not answered** |
| Diagnosis of ID |  |  |  |  |  |
| IDA (n=109) | 0 (0.0) | 0 (0.0) | 46 (42.2) | 61 (56.0) | 2 (1.8) |
| IDNA (n=192) | 4 (2.1) | 2 (1.0) | 48 (25.0) | 138 (71.9) | 0 (0.0) |
| Cause of ID |  |  |  |  |  |
| HMB (n=147) | 0 (0.0) | 0 (0.0) | 27 (18.4) | 120 (81.6) | 0 (0.0) |
| Other (n=125) | 2 (1.6) | 2 (1.6) | 58 (46.4) | 62 (49.6) | 1 (0.7) |
| Concurrent disease |  |  |  |  |  |
| Yes (n=134) | 1 (0.7) | 2 (1.5) | 55 (41.0) | 75 (56.0) | 1 (0.7) |
| No (n=167) | 3 (1.8) | 0 (0.0) | 39 (23.4) | 124 (74.3) | 1 (0.6) |
| Concomitant medication/therapeutic intervention |  |  |  |  |  |
| Yes (n=110) | 1 (0.9) | 0 (0.0) | 49 (44.5) | 59 (53.6) | 1 (0.9) |
| No (n=191) | 3 (1.6) | 2 (1.0) | 45 (23.6) | 140 (73.3) | 1 (0.5) |

HMB, heavy menstrual bleeding; ID, iron deficiency; IDA, iron deficiency anemia; IDNA, iron deficiency without anemia.

## Supplementary Table S12

Baseline demographic and clinical characteristics of patients by overall level of patient satisfaction with ferric derisomaltose treatment at baseline

| **n (%)** | **Patient satisfaction at baseline** | | | | |
| --- | --- | --- | --- | --- | --- |
|  | **Very dissatisfied** | **Dissatisfied** | **Satisfied** | **Very satisfied** | **Not answered** |
| Cause of ID |  |  |  |  |  |
| HMB (n=146) | 4 (2.7) | 2 (1.4) | 20 (13.7) | 87 (59.6) | 33 (22.6) |
| Other (n=127) | 4 (3.1) | 4 (3.1) | 57 (44.9) | 41 (32.3) | 21 (16.5) |
| Concurrent disease |  |  |  |  |  |
| Yes (n=134) | 4 (3.0) | 5 (3.7) | 50 (37.3) | 46 (34.3) | 29 (21.6) |
| No (n=168) | 5 (3.0) | 2 (1.2) | 31 (18.5) | 103 (61.3) | 27 (16.1) |
| Concomitant medication/therapeutic intervention |  |  |  |  |  |
| Yes (n=110) | 2 (1.8) | 5 (4.5) | 42 (38.2) | 38 (34.5) | 23 (20.9) |
| No (n=192) | 7 (3.6) | 2 (1.0) | 39 (20.3) | 111 (57.8) | 33 (17.2) |

HMB, heavy menstrual bleeding; ID, iron deficiency.

## Supplementary Table S13

Baseline demographic and clinical characteristics of patients by overall level of physician satisfaction with ferric derisomaltose treatment at follow-up

| **n (%)** | **Physician satisfaction at follow-up** | | | | |
| --- | --- | --- | --- | --- | --- |
|  | **Very dissatisfied** | **Dissatisfied** | **Satisfied** | **Very satisfied** | **Not answered** |
| Diagnosis of ID |  |  |  |  |  |
| IDA (n=91) | 2 (2.2) | 1 (1.1) | 41 (45.1) | 46 (50.5) | 1 (1.1) |
| IDNA (n=176) | 1 (0.6) | 4 (2.3) | 48 (27.3) | 122 (69.3) | 1 (0.6) |
| Cause of ID |  |  |  |  |  |
| HMB (n=128) | 0 (0.0) | 1 (0.8) | 37 (28.9) | 90 (70.3) | 0 (0.0) |
| Other (n=117) | 3 (2.6) | 4 (3.4) | 46 (39.3) | 63 (53.8) | 1 (0.9) |
| Concurrent disease |  |  |  |  |  |
| Yes (n=123) | 2 (1.6) | 2 (1.6) | 52 (42.3) | 66 (53.7) | 1 (0.8) |
| No (n=144) | 1 (0.7) | 3 (2.1) | 37 (25.7) | 102 (70.8) | 1 (0.7) |

HMB, heavy menstrual bleeding; ID, iron deficiency; IDA, iron deficiency anemia; IDNA, iron deficiency without anemia.

## Supplementary Table S14

Baseline demographic and clinical characteristics of patients by overall level of patient satisfaction with ferric derisomaltose treatment at follow-up

| **n (%)** | **Patient satisfaction at follow-up** | | | | |
| --- | --- | --- | --- | --- | --- |
|  | **Very dissatisfied (n=7)** | **Dissatisfied (n=10)** | **Satisfied (n=94)** | **Very satisfied (n=149)** | **Not answered (n=5)** |
| Diagnosis of ID |  |  |  |  |  |
| IDA (n=92) | 3 (3.3) | 4 (4.3) | 42 (45.7) | 39 (42.4) | 4 (4.3) |
| IDNA (n=173) | 4 (2.3) | 6 (3.5) | 52 (30.1) | 110 (63.6) | 1 (0.6) |
| Cause of ID |  |  |  |  |  |
| HMB (n=122) | 2 (1.6) | 1 (0.8) | 32 (26.2) | 84 (68.9) | 3 (2.5) |
| Other (n=121) | 3 (2.2) | 8 (5.8) | 56 (46.3) | 52 (43.0) | 2 (1.7) |
| Concurrent disease |  |  |  |  |  |
| Yes (n=126) | 4 (3.2) | 2 (1.6) | 60 (47.6) | 58 (46.0) | 2 (1.6) |
| No (n=139) | 3 (2.2) | 8 (5.8) | 34 (24.5) | 91 (65.5) | 3 (2.2) |
| Concomitant medication/therapeutic intervention |  |  |  |  |  |
| Yes (n=107) | 4 (3.7) | 3 (2.8) | 49 (45.8) | 49 (45.8) | 2 (1.9) |
| No (n=158) | 3 (1.9) | 7 (4.4) | 45 (28.5) | 100 (63.3) | 3 (1.9) |

HMB, heavy menstrual bleeding; ID, iron deficiency; IDA, iron deficiency anemia; IDNA, iron deficiency without anemia.

**References**

1. European Medicines Agency (2024) Guideline on good pharmacovigilance practices (GVP) Annex I - Definitions (Rev 5). <https://www.ema.europa.eu/en/documents/regulatory-procedural-guideline/guideline-good-pharmacovigilance-practices-gvp-annex-i-definitions-rev-5-track-changes_en.pdf>. Accessed 7 August 2024
